# Modernising patient education in vitreoretinal surgery

**DOI:** 10.1038/s41433-025-04206-1

**Published:** 2026-01-09

**Authors:** Jae Yee Ku, Rumana Hussain, Heinrich Heimann, Nima Ghadiri, Ivan Sychev, Max Griebsch, Aruni Makuloluwa, Christopher Ze Qian Go, Konstantinos Kaprinis, Tom Southern, Keeran Shou Yao Chang, Paul Y. Chua, Carl Groenewald, Fidan Jmor, Ian Pearce, Teresa Sandinha, Sandra Gray, Shi Zhuan Tan

**Affiliations:** 1 Department of Eye and Vision Science, Institute of Life Course and Medical Sciences, Faculty of Health & Life Sciences, University of Liverpool, William Henry Duncan Building, Liverpool, UK; 2 Royal Liverpool University Hospital, Liverpool, UK; 3 Faculty of Medicine, University of New South Wales, Wallace Wurth Building, Sydney, NSW Australia; 4 Save Sight Institute, University of Sydney, Sydney, NSW Australia; 5 Brisbane Grammar School, Spring Hill, QLD Australia

**Keywords:** Education, Health services

## Introduction

Poor literacy is a major barrier to effective healthcare communication, with one in six people in the UK affected [1]. In Liverpool, literacy vulnerability is among the highest in the North West of England [2, 3]. At the same time, social media has shaped patient preferences, with images and videos now the preferred modes of learning and engagement. Traditional, text-heavy patient education materials risk leaving many patients behind. In vitreoretinal surgery, this gap has significant clinical consequences. Patients often struggle to understand written postoperative instructions or read small print, particularly those relating to postoperative posturing. Poor adherence can compromise surgical success and lead to poorer visual outcomes [4, 5].

To address this, we developed a new patient information leaflet (PIL) that incorporates clear illustrations and is complemented by short instructional videos. This quality improvement project aims to enhance patient understanding of posturing instructions after vitreoretinal surgery. By modernising educational resources, this initiative promotes patient-centred care in the Royal Liverpool University Hospital and was delivered using the Model for Improvement through a Plan-Do-Study-Act (PDSA) cycle described below [6, 7].

## Stage 1: plan

A baseline questionnaire was completed by staff for day one postoperative patients to assess their understanding of postoperative care, including proper posturing. The questionnaire included three questions (Table 1), each answered on a five-point Likert scale (very certain, certain, neutral, uncertain, very uncertain). Of the 20 questionnaires collected over two weeks, most patients (85%) felt very certain or certain that posturing was required. However, only 60% reported being very certain or certain about the correct posture itself, and fewer still (45%) were confident about the duration for which posturing should be maintained (Table 1). In response to these findings, we proposed a new patient information leaflet (PIL) featuring illustrations of key postures and embedded QR codes linking to instructional videos.

**Table 1:** Questionnaire of patients’ certainty of their postoperative posturing instructions before and after the introduction of the new patient information leaflet (PIL).

Questions	Very certain/Certain		Neutral		Uncertain/Very uncertain	
	Before PIL	After PIL	Before PIL	After PIL	Before PIL	After PIL
**1. How certain is the patient that they need to posture?**	85% (17/20)	94.4% (17/18)	5% (1/20)	0% (0/18)	10% (2/20)	5.6% (1/18)
**2. How certain is the patient of their posture?**	60% (12/20)	83.3% (15/18)	20% (4/20)	5.6% (1/18)	20% (4/20)	11.1% (2/18)
**3. How certain is the patient of their posturing duration**	45% (9/20)	72.2% (13/18)	10% (2/20)	5.6% (1/18)	45% (9/20)	22.2% (4/18)

## Stage 2: do

**Fig. 1:**
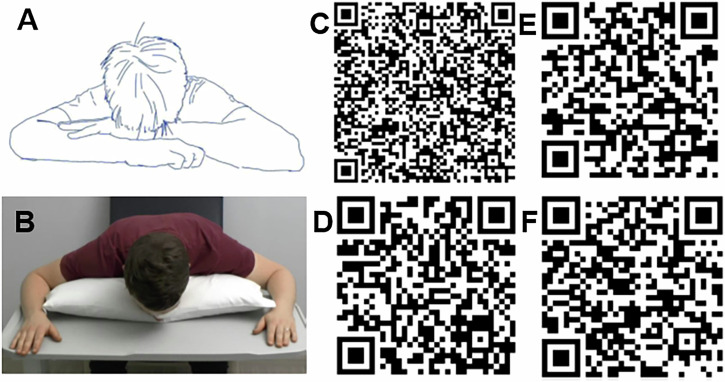
Figures and QR codes shown in the patient information leaflet. Example of the face down posture illustrated in the patient information leaflet (**A**) and demonstrated in the video (**B**). QR codes to obtain the patient information leaflet (**C**) nd view the videos for posturing after macular hole surgery (**D**), retinal detachment surgery (**E**) and the technique for applying eye drops (**F**).

A local team convened to develop the new PIL incorporating illustrations of key postoperative postures. To complement this, three educational videos were produced covering posturing after macular hole surgery, after retinal detachment surgery and the correct technique for instilling postoperative eye drops. These videos were launched on the trust’s YouTube channel in April 2024. Staff in the theatre admission unit who care for postoperative patients received training on how to implement the new PIL. To enhance accessibility for individuals with visual impairment, the PIL has a minimum 16-point font and is printed on yellow paper, as recommended by the Royal National Institute of Blind People (RNIB) [8]. The PIL and videos could be viewed by scanning the QR codes in Fig. 1.

## Stage 3: study

Two months after introducing the new PIL, we reassessed its impact on patients’ certainty regarding posturing instructions. The original questionnaire was repeated over two weeks (*n* = 18). Compared with baseline, the proportion of patients who were very certain or certain increased to 94.4% for Question 1, 83.3% for Question 2, and 72.2% for Question 3 (Table 1).

We obtained more specific patient feedback in a questionnaire with the following 5 questions (*n* = 8). Feedback (indicated in brackets) was uniformly positive.

1. Do you feel that there is a need for this PIL? (Yes, 100%)
2. Do you feel that the amount of information (As much as you want, 100%)
3. Did you find the information in the leaflet clear to understand? (Understood all the information, 100%)
4. Do you think the size of the print is (Just right, 100%)
5. Do you think the information on each page is (Just right, 100%)

In addition, staff feedback was collected using the following 5-question (*n* = 8) questionnaire. Responses (indicated in brackets) were strongly supportive of the new PIL.

1. How satisfied are you overall with the PIL? (Very satisfied 87.5%, Satisfied 12.5%, Neutral 0%, Unsatisfied 0%, Very unsatisfied 0%)
2. How easy is it to complete the PIL? (Very easy 87.5%, Easy 12.5%, Neutral 0%, Difficult 0%, Very difficult 0%)
3. How much time does it take to complete the PIL (Very short 50%, Short 50%, Neutral 0%, Long 0%, Very long 0%)
4. How certain are you that the new PIL will help patients understand their posturing instructions better? (Very certain 37.5%, Certain 50%, Neutral 12.5%, Uncertain 0%, Very uncertain 0%)
5. Is the PIL available for you to use? (Very available 62.5%, Available 25%, Neutral 12.5%, Unavailable 0%, Very unavailable 0%)

## Stage 4: act

Based on the questionnaire results and feedback, the current format of the PIL was deemed satisfactory. In addition to the planned formal review of the PIL in three years, the highly viewed patient instructional videos (over 22,000 views for the retinal detachment video; over 15,000 views for the macular hole video) available on the trust’s YouTube channel will be maintained and promoted as a sustainable, internationally accessible tool. As part of this effort, the videos have been made available on the British & Eire Association of Vitreoretinal Surgeons (BEAVRS) website as a patient resource [9].

## Conclusion

This quality improvement project successfully achieved its aim of modernising patient education in vitreoretinal surgery. By introducing a visually enhanced PIL supported by instructional videos, we improved patients’ certainty in understanding and following their postoperative instructions. Both patient and staff feedback were overwhelmingly positive, confirming the value of clear, accessible, and engaging educational resources. Interestingly, the countries that have viewed these videos the most are the USA and India, suggesting that these videos are having an impact beyond the UK. This work demonstrates that adapting patient information to contemporary learning preferences can significantly enhance patient-centred care, and it provides a sustainable model for future innovations in patient education.

## Data Availability

The datasets generated during and/or analysed during the current study are available from the corresponding author on reasonable request.
